# A case of intestinal fistula following surgery for a large ovarian tumor complicated by abdominal tuberculosis: a case report and review of the literature

**DOI:** 10.1186/s13256-025-05744-8

**Published:** 2025-12-11

**Authors:** Qiang Ji

**Affiliations:** Department of Gynecology, Affiliated Zhongshan hospital of Dalian university, Dalian, 116000 China

**Keywords:** Case report, Ovarian tumor, Abdominal tuberculosis, Intestinal fistula

## Abstract

**Background:**

Abdominal tuberculosis is a rare and diagnostically challenging form of extrapulmonary tuberculosis that can closely mimic advanced ovarian cancer both clinically and radiologically. This case is reported to highlight this diagnostic dilemma and the serious postoperative complications that can arise, to enhance clinical awareness and reduce misdiagnosis.

**Case presentation:**

A 68-year-old female of Asian ethnicity presented with a 1-year history of heartburn and significant weight loss. Preoperative evaluation revealed a large pelvic mass, elevated cancer antigen-125 (309.2 U/mL), and ascites, highly indicative of ovarian malignancy. She underwent laparoscopic exploration converted to laparotomy for left adnexectomy owing to extensive adhesions. Postoperative pathology confirmed a benign ovarian mucinous cystic adenoma and, unexpectedly, necrotic granulomatous tissue was observed in the abdominal wall tissue and mesosalpinx, consistent with tuberculosis. A total of 2 weeks post surgery, the patient developed an enterocutaneous fistula, which was managed conservatively with targeted antituberculosis therapy, prolonged drainage, and nutritional support. After over a year of treatment, the fistula healed completely and the patient regained weight, achieving a full recovery.

**Conclusion:**

This case underscores the importance of considering abdominal tuberculosis in the differential diagnosis of ovarian cancer, especially in endemic areas. A high index of suspicion, utilizing a combination of diagnostic tools, and careful intraoperative assessment are crucial. Once diagnosed, conservative management with sustained antituberculosis therapy and nutritional support can lead to excellent outcomes even for complex complications such as intestinal fistula, avoiding the need for high-risk reoperation.

## Background

Tuberculosis (TB) is an infectious disease caused by *Mycobacterium tuberculosis*, with approximately 7.5 million new cases diagnosed in 2022, and affects primarily the lungs but may also affect other organs, referred to as extrapulmonary TB [1]. Abdominal TB accounts for approximately 10% of all extrapulmonary cases [2]. The main routes of infection include gastrointestinal infection, hematogenous, and lymphatic dissemination. In this article, we report a case of a patient with suspected ovarian malignant tumor that was diagnosed as benign ovarian tumor combined with abdominal tuberculosis by postoperative pathology.

## Case presentation

**Fig. 1:**
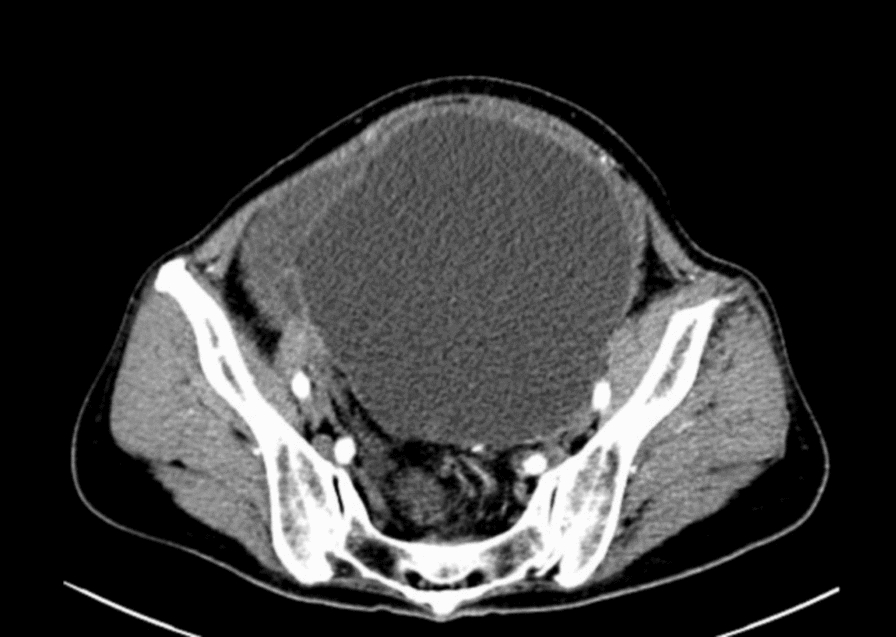
Preoperative axial computed tomography scan of the pelvis showing a large cystic mass and associated ascites

The patient was a 68-year-old Asian female who presented with a 1-year history of heartburn and weight loss. Her weight was 43 kg, with a body mass index (BMI) of 16.8 kg/m^2^. The patient’s past medical history was unremarkable, with no significant chronic illnesses, infectious diseases, prior surgeries, or allergies. Family history was noncontributory for malignancies, tuberculosis, or hereditary disorders. She denied any history of smoking or alcohol use. There was no recent travel to tuberculosis-endemic areas or known contact with individuals with active TB. Prior to this presentation, the patient had not received any pharmacological or surgical interventions for these symptoms. Abdominal computed tomography (CT) revealed a 15 cm pelvic cystic mass with associated peritoneal effusion, raising concerns about primary peritoneal malignancy or peritoneal metastasis (Fig. 1). Cancer antigen (CA)-125 was 309.2 U/mL (0–30.2U/mL), HE4 was 92.3 pmol/L (premenopausal: < 74.05/postmenopausal: < 147.75 pmol/L), and hemoglobin was 95 g/L. Chest CT showed no significant abnormalities. On gynecological examination, the vaginal canal was patent. The cervix was of normal size and smooth. A large, immobile cystic mass was palpable in the lower abdomen, raising suspicion of ovarian malignancy. A digital rectal examination revealed that the rectal mucosa was smooth. The uterosacral ligaments showed no significant thickening or shortening. The examining glove was withdrawn without blood stains.

**Fig. 2:**
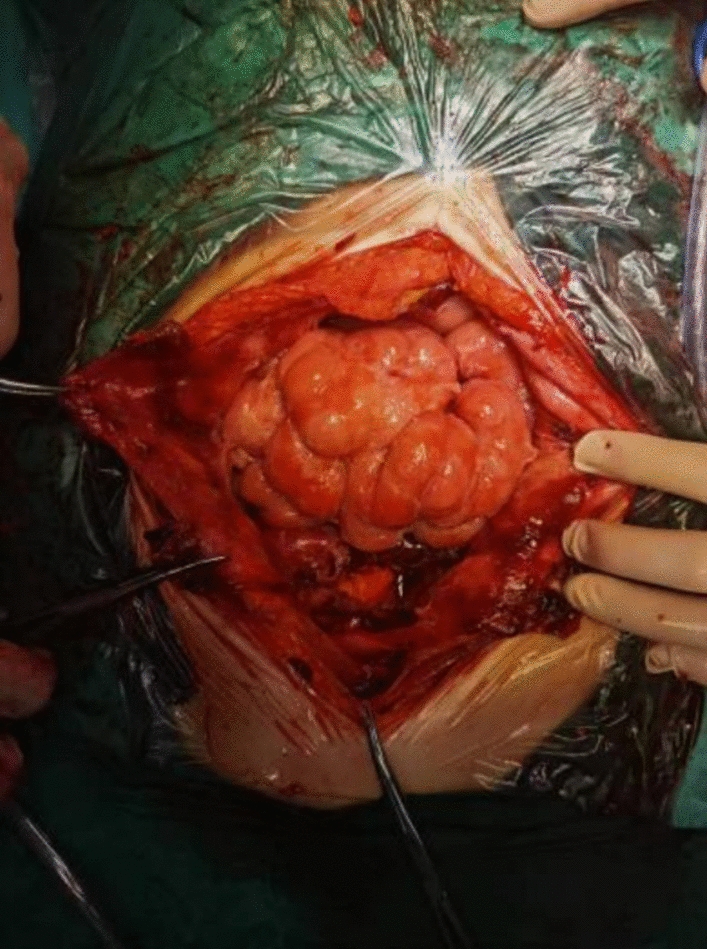
Intraoperative photograph showing extensive filamentous adhesions on the surface of the intestinal tract

The patient subsequently underwent laparoscopic exploration. Intraoperatively, we observed a left-sided ovarian cystic mass measuring approximately 15 cm in diameter. Extensive mesh-like and membranous adhesions were present on the abdominal and pelvic walls, with dense adhesions fixing the posterior aspect of the mass to the pelvic floor. Given the severity of these adhesions, the procedure was converted to open laparotomy to ensure complete tumor resection and minimize the risk of injury. Upon continuing the exploration via laparotomy, we noted that the left fallopian tube was thickened and the mesentery had a pancake-like appearance. The left fallopian tube and sigmoid colon were densely adherent to the cyst surface, severely compressing and flattening the intestinal lumen. Most of the appendices epiploicae appeared atrophied. The abdominal and pelvic peritoneum were markedly thickened, with multiple areas of purulent and necrotic change. The surfaces of the abdominal and pelvic organs, as well as the peritoneum and intestines, exhibited varying degrees of inflammatory exudate, and filamentous adhesions formed fibrous, band-like structures (Fig. 2).

After separating the adhesions, the left ovary and fallopian tube were completely resected and sent for intraoperative frozen pathologic examination, suggesting a benign cystic adenoma. Owing to severe localized adhesions and the poor nutritional status of the intestinal wall, an intraoperative surgical consultation was obtained. The consultant noted no obvious intestinal breakage or obstruction, and blood flow to the bowel appeared adequate, as there was no evidence of an enterotomy, integrity tests were not performed to minimize manipulation of the fragile, adherent bowel. The surgery was then successfully concluded. The patient was fasted for 1 week after surgery, and was given parenteral nutrition with enough calories and albumin. She had irregular fever up to 39 ℃, which improved and stabilized after anti-inflammatory and symptomatic treatment, and the pelvic drainage tube was removed on the ninth day after surgery.

Postoperative pathological examination confirmed a benign ovarian mucinous cystic adenoma, and necrotic granulomatous tissue was seen in the abdominal wall tissue and mesosalpinx, which was considered to be tuberculosis. To further support the diagnosis, a tuberculin skin test and an interferon-gamma release assay were conducted, with both results being positive. Unfortunately, microbiological confirmation—such as acid-fast bacilli (AFB) smear, mycobacterial culture, or molecular testing like GeneXpert MTB/RIF—was not performed, as specimens were not routinely collected for such purposes during the surgery. She was discharged on postoperative day 14 and was advised to seek further diagnosis and treatment at a specialized tuberculosis hospital.

**Fig. 3:**
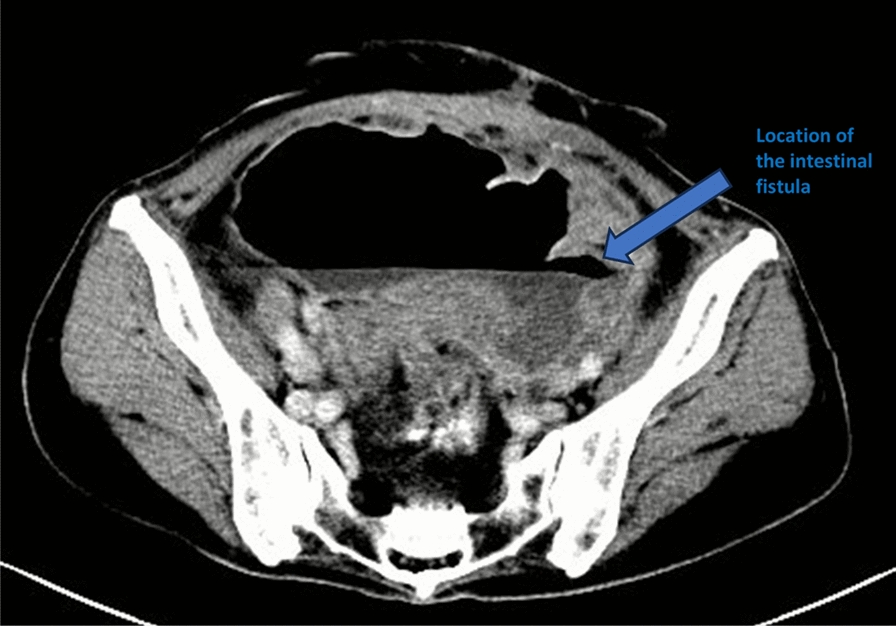
Postoperative computed tomography image demonstrates a localized fluid collection and extraluminal air secondary to an enteric fistula, with the arrow indicating the site of the fistula

**Fig. 4:**
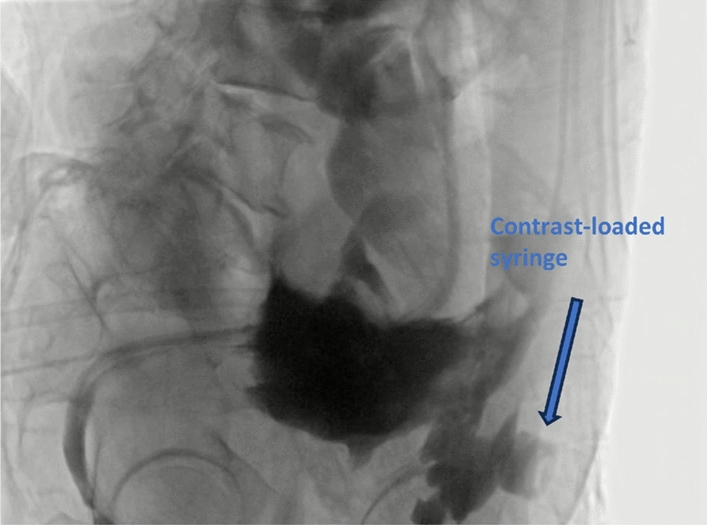
Contrast agent was injected via the transabdominal sinus tract, opacifying the local cavity formed by the enterocutaneous fistula. The arrow indicates the contrast-loaded syringe

The patient developed a sudden high fever of 39 ℃ that evening. An abdominal enhanced CT suggested a large amount of fluid accumulation in the abdominopelvic cavity, and perforation of the sigmoid colon with localized encapsulation and localized dilatation of the sigmoid colon was considered (Fig. 3). Perforation was seen at the descending sigmoid junction by intestinal imaging under intervention, and contrast was seen in the pelvis, with severe dilatation of the whole colon; pelvic encapsulated fluid was punctured and drained, which yielded dark brown dilute stool. In addition, dilute stool and a large amount of gas were drained through the anorectal tube. On the second postoperative day, the transabdominal incision exuded yellow–green loose stool, and the incision was examined by incision imaging under intervention, which showed that the incision was connected with the local cystic lumen (Fig. 4). The drainage tube was adjusted to ensure patency.

Octreotide (a somatostatin analog) was administered intravenously at 0.24 mg/hour for 5 days, then reduced to 0.12 mg/hour for 2 days, to inhibit intestinal fluid secretion. The patient was managed with fasting, fluid resuscitation, anti-inflammatory therapy, systemic support, and local irrigation and drainage. After consultation at the tuberculosis hospital, antituberculosis treatment was given (isoniazid 0.3 g once daily intravenously; moxifloxacin 0.4 g once daily intravenously; amikacin 0.4 g once every 12 hours intravenously) and the patient’s condition stabilized after more than 1 month of treatment, the catheter for intestinal obstruction was removed, the feces from the incision did not seep out irregularly, and the diet was gradually resumed, with bowel and urinary function returning to normal. The regimen was then switched to oral antituberculosis therapy (rifampicin 0.45 g once daily, isoniazid 0.3 g once daily, pyrazinamide 0.5 g twice daily, and ethambutol 0.5 g once daily).

**Fig. 5:**
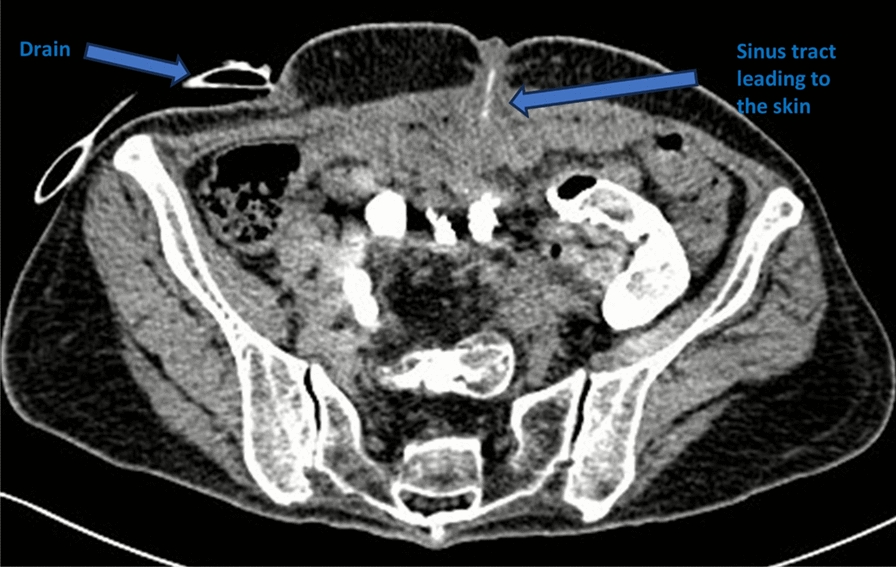
The computed tomography scan reveals an established enterocutaneous fistula (right-sided arrow) with an *in situ* drainage tube also visible (left-sided arrow)

**Fig. 6:**
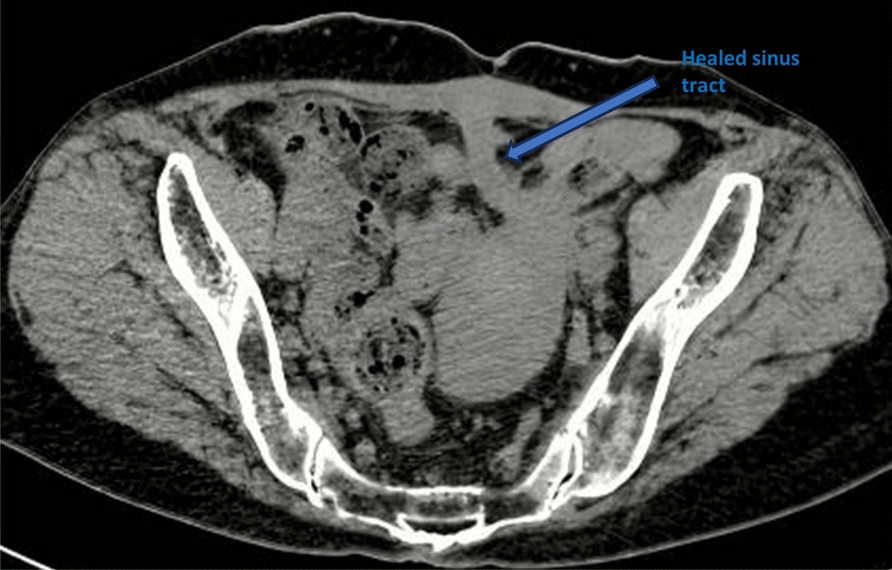
Following 1.5 years of treatment, the sinus tract is completely healed, as indicated by the arrow

The patient was discharged from the hospital and continued to recover at home; the abdominal drainage was washed and drained every day to maintain patency, the incision oozing was disinfected and changed on a regular basis, the antituberculosis treatment was continued, and the nutrition was actively strengthened (Fig. 5). After 1.5 years, the patient’s abdominal drainage was gradually reduced and withdrawn and the abdominal incision oozing was gradually reduced and healed (Fig. 6). The patient reported that her appetite and diet had improved significantly compared with that before the onset of the disease, and her weight had increased to 55 kg. The patient’s clinical course is presented in Table 1.

**Table 1:** Clinical timeline of presentation, management, and follow-up

Time point (relative to admission)	Key clinical events and interventions
*T* = −12 months	Onset of heartburn and progressive weight loss
*T* = 0 (admission)	Hospital admission. CT reveals a 15-cm pelvic mass, ascites, and elevated CA-125 (309.2 U/mL). Highly indicative of ovarian malignancy
*T* = +3 days	Laparoscopic exploration, converted to open laparotomy (left adnexectomy) owing to severe adhesions
*T* = +14 days	Discharged. Final pathology confirms benign ovarian mucinous cystic adenoma and necrotic granulomas consistent with tuberculosis
*T* = +14 days (evening)	Readmission with sudden high fever (39 °C). CT suggests sigmoid colon perforation and localized encapsulation
*T* = +15 days	Enterocutaneous fistula confirmed by fistulography. Initiation of conservative management: Anti-TB therapy (isoniazid, moxifloxacin, amikacin IV), fasting, total parenteral nutrition, and active drainage
*T* = +2 months	Condition stabilized. Switched to oral anti-TB regimen (rifampicin, isoniazid, pyrazinamide, ethambutol). Gradual resumption of diet
*T* = +1.5 years	Fistula completely healed. Abdominal drainage tube removed. Incision site fully closed. Weight increased to 55 kg. Full recovery of bowel and urinary function

## Discussion and conclusion

From a gynecological perspective, the patient’s pelvic mass with elevated CA-125 levels and peritoneal changes on imaging initially suggested ovarian cancer. However, this case highlights the importance of considering abdominal tuberculosis, especially in the absence of definitive ovarian tumor tissue, underscoring the need for thorough preoperative and differential diagnoses.

Common symptoms of abdominal tuberculosis include abdominal pain (95%), followed by weight loss (88%), fever (84.6%), abdominal mass (46.1%), and a range of other symptoms including vomiting, constipation, abdominal pressure, ascites, and peritonitis [3]. However, these symptoms are usually not specific and are often similar in patients with ovarian cancer, making it difficult to differentiate between the two on the basis of symptoms alone.

Conventional hematological tests are inexpensive and readily available, and although anemia and elevated blood sedimentation are nonspecific, they are suggestive of the need for further testing or a clinical differential diagnosis [4, 5]. In this case, the patient was mildly anemic before surgery, and after the diagnosis was confirmed, the erythrocyte sedimentation rate (ESR) was 78 mm/hour, which gradually decreased to normal during the treatment and follow-up.

In laboratory tests, CA-125 is not discriminatory and is elevated in a wide range of diseases and conditions. It is also frequently elevated in patients with abdominal tuberculosis. For instance, a study by Maheshwari *et al*. [6] involving 120 patients with abdominal tuberculosis reported a mean CA-125 level of 666.9 (range 38–18554) U/mL. However, CA-125 has some significance in evaluating the efficacy of therapy and the activity of tuberculosis after treatment of abdominal tuberculosis [7].

Human epididymis protein 4 (HE-4) is also elevated to some extent in patients with tuberculosis, Zhang *et al*. [8] concluded that HE-4 of 151.4 pmol/L can be used as a critical value to differentiate abdominal tuberculosis from ovarian cancer.

The gamma-interferon release assay offers higher sensitivity and specificity than the tuberculin test. However, positive results should be interpreted in conjunction with specific clinical symptoms to differentiate between latent and active tuberculosis. A study [9] showed that gamma-interferon release assay in peripheral blood of tuberculous peritonitis has a sensitivity of 55.6%, a specificity of 92.3%, a diagnostic efficiency of 77.3%, and a diagnostic efficiency of 81.8% in ascites.

Conventional smear microscopy is the classic TB test but has low sensitivity, more false negative results, and is affected by subjective factors and specimen quality, which was shown to be 0–40% in one study. Mycobacterial culture can confirm the diagnosis of TB, and further drug sensitivity testing is possible, but the culture time is long, usually 2–8 weeks, and the detection rate varies from 30% to 80% [10].

Gene Xpert MTB/RIF assay is an assay based on the principle of real-time quantitative fluorescence polymerase chain reaction (PCR), which can detect the presence of *Mycobacterium tuberculosis* and drug resistance in samples within 2 hours. It is one of the fastest molecular diagnostic techniques for tuberculosis, which can accurately exclude nontuberculous mycobacteria and identify rifampicin resistance; it is simple to operate, highly accurate, and has a low probability of contamination. However, it is expensive, difficult to promote commercially, and is not sensitive enough for patients with paucibacillary TB and human immunodeficiency virus (HIV). On the basis of this, Gene Xpert MTB/RIF Ultra adds two additional molecular targets (IS1081 and IS6110) for *Mycobacterium tuberculosis* (MTB) detection to increase the sensitivity of the assay. In a prospective study [11] that included 225 cases, including 200 extrapulmonary tuberculosis (EPTB) and 25 non-EPTB cases, the sensitivities of Xpert Ultra and Xpert for culture-positive cases were 83.7% and 67.4%, respectively. Specificity was 92.0% and 96.0%, respectively. The sensitivities of Xpert Ultra, Xpert, and *M. tuberculosis* cultures for 200 EPTB cases were 52.5%, 34.0%, and 21.5%, respectively. However, Xpert MTB Ultra could not differentiate the activity of *M. tuberculosis* and has limitations in diagnosis in patients with a history of tuberculosis.

Adenosine deaminase (ADA) is an important enzyme in the process of purine adenosine catabolism and metabolism, and its content is highest in T lymphocytes and is positively proportional to the degree of differentiation of T lymphocytes. When T lymphocytes are stimulated by *Mycobacterium tuberculosis* antigens, the activity of ADA in peritoneal fluid is increased [12].The activity of ADA in peritoneal fluid increased when T lymphocytes were stimulated by *Mycobacterium tuberculosis* antigen. In a meta-analysis [13], the sensitivity and specificity of ascites ADA for the diagnosis of tuberculous peritonitis were found to be 0.93 (95% confidence interval [CI] 0.89–0.95) and 0.96 (95% CI 0.94–0.97), respectively, with an area of 0.98 under the summary receiver operating characteristic curve (SROC).

On imaging, abdominal tuberculosis is often difficult to differentiate from progressive ovarian cancer and peritoneal metastases on CT [14]. However, it has been suggested in some studies that the peritoneum tends to show smooth thickening in abdominal tuberculosis, whereas in malignant tumors, the peritoneum often shows irregular nodular thickening [15–17]. In another study, it was suggested that patients with peritoneal carcinomatosis more commonly had inhomogeneous substantial thickening and peritoneal changes in the ovaries compared with abdominal tuberculosis, with no statistically significant difference in other CT features [18].

Positron emission tomography (PET)–CT, as a functional metabolic imaging method, carries out diagnosis through [18] F-deoxyglucose (FDG) metabolism differences between diseased and normal tissues, but it is also because of its special working principle that both abdominal tuberculosis and malignant tumors show high metabolism in PET–CT, so it is difficult to differentiate between the two. However, PET–CT can be used for patients with confirmed diagnosis of abdominal tuberculosis to evaluation of disease and treatment effect [6, 19, 20].

When laboratory tests and imaging examinations fail to make a clear diagnosis, tissue biopsy under laparoscopic surgery is an effective diagnostic method [21, 22]. According to relevant studies, the diagnostic rate of laparoscopic tissue biopsy is 85–95%, and abdominal tuberculosis is often characterized by free ascites with multiple yellowish–white nodules, visceral or peritoneal nodules, peritoneal and visceral adhesions, and scattered inflammatory hemorrhages in the peritoneum [5]. However, it is important to note that in adhesive peritonitis, the risk of iatrogenic injury to adjacent structures is higher due to extensive visceral adhesions due to massive fibrous tissue proliferation and conversion to open surgery to separate the adhesions if necessary [2].

Owing to the extreme lack of intestinal nutrition explored in this patient intraoperatively and the patient’s wasting presentation, we realized that postoperative nutrition was also extremely important for her recovery [1, 26]. Despite adequate postoperative nutrition and a long enough fast, we were unable to prevent this outcome. Combined with the fact that the patient had an intestinal obstruction in the presence of an enterocutaneous fistula, we believe that the patient had postoperative adhesions that caused intestinal obstruction, which led to dilatation of the intestinal tube, and poor nutritional state of the intestinal tube, which then led to perforation of the intestinal tube at the weak point. However, abdominal tuberculosis can cause spontaneous intestinal perforation, In one study, 45 patients with abdominal tuberculosis were reported, and 9 patients underwent emergency surgery for bowel perforation, which can greatly exacerbate the condition and increase the mortality rate [23]. Fortunately, in our case, adhesions and encapsulation formed around the fistula, which prevented the development of severe peritonitis and prevented serious infection from the onset of the fistula to its healing, and the formation of a drainage channel through the abdominal wall made it possible to treat the patient conservatively with localized drainage. The current surgical consensus is to consider surgical repair after 3–6 months of failed conservative management for enterocutaneous fistulas, primarily for infection control and nutritional improvement [24, 25]. We also considered surgical treatment. However, in the present case, a conservative approach extending beyond this timeframe was pursued for several reasons. First, the underlying etiology of the fistula was active abdominal TB, the control of which and the subsequent resolution of peritoneal inflammation is a protracted process. Operating before the infection is adequately controlled carries a high risk of failure and complications. Second, despite the prolonged time to complete closure, the patient’s general condition improved steadily under anti-TB therapy, with a consistent reduction in fistula output, indicating a positive trend that obviated the need for emergent surgery. Finally, considering the patient’s formation of a complex fistula, the possibility of secondary injury due to separation of adhesions, the patient’s poor nutritional status, and the possibility of postoperative anastomotic fistula, we opted for conservative treatment and then considered surgical treatment if there was no tendency for improvement. Through local drainage and concomitant antituberculosis treatment, the drainage from the abdominal wall fistula gradually decreased and the fistula eventually healed completely after 1.5 years of treatment. The 18-month antituberculosis course in this case exceeded the standard 6-month regimen recommended by the World Health Organization [1]. This extended duration was necessitated by the patient’s presentation with a complex enterocutaneous fistula, extensive abdominal adhesions, and severe pre-existing malnutrition. Furthermore, the patient demonstrated good tolerance to the anti-TB medications without significant adverse effects. Therefore, in consultation with an infectious disease specialist, the decision was made to extend the treatment until complete clinical and radiological resolution was achieved, ensuring a full recovery.

In addition to ovarian malignancy, several other diseases can present with similar clinical and radiologic findings to abdominal tuberculosis and should be carefully differentiated. Peritoneal carcinomatosis secondary to gastrointestinal malignancies may also manifest with ascites and peritoneal thickening; however, in this patient, gastrointestinal endoscopy revealed no evidence of a primary gastrointestinal tumor and postoperative histopathology showed granulomatous inflammation without malignant cells. Crohn’s disease can lead to transmural intestinal inflammation and fistula formation, but the patient had no history of chronic diarrhea, hematochezia, or abdominal pain, and the biopsy lacked the noncaseating granulomas characteristic of Crohn’s disease. Sarcoidosis and other granulomatous diseases were excluded because there were no signs of systemic involvement such as pulmonary or lymph node lesions and the patient showed a favorable response to antituberculosis therapy. Taken together, these findings supported abdominal tuberculosis as the most likely underlying etiology [16, 27].

In conclusion, the possibility of abdominal tuberculosis should be considered in any patient with suspected ovarian cancer, and the diagnosis should be made progressively through laboratory and imaging tests, with laparoscopic tissue biopsy for definitive diagnosis when the diagnosis is unclear, and open abdominal exploration when necessary. The patient’s postoperative nutrition is likewise crucial and has an important impact on patient prognosis.

## Data Availability

The datasets used and/or analyzed during the current study are available from the corresponding author on reasonable request.

## Abbreviations

TB: Tuberculosis
ADA: Adenosine deaminase
FDG: F-deoxyglucose
HE4: Human epididymis protein 4
